# Detection of Surface Defects in Steel Based on Dual-Backbone Network: MBDNet-Attention-YOLO

**DOI:** 10.3390/s25154817

**Published:** 2025-08-05

**Authors:** Xinyu Wang, Shuhui Ma, Shiting Wu, Zhaoye Li, Jinrong Cao, Peiquan Xu

**Affiliations:** 1 School of Materials Science and Engineering, Shanghai University of Engineering Science, Shanghai 201620, China; 18761011210@163.com (X.W.); 18501684828@163.com (S.W.); hmcaojinrong@163.com (J.C.); 2 School of Arts and Sciences, Northeast Agricultural University, Harbin 150030, China; 15146407953@163.com; 3 School of Lilac, Harbin Institute of Technology (Weihai), Weihai 264209, China; fc_12enghi@163.com; 4 Shanghai Collaborative Innovation Center of Laser Advanced Manufacturing Technology, Shanghai University of Engineering Science, Shanghai 201620, China

**Keywords:** MBDNet, YOLO, dynamic align fusion, MultiSEAM, Inner-SIoU, steel surface defect detection

## Abstract

Automated surface defect detection in steel manufacturing is pivotal for ensuring product quality, yet it remains an open challenge owing to the extreme heterogeneity of defect morphologies—ranging from hairline cracks and microscopic pores to elongated scratches and shallow dents. Existing approaches, whether classical vision pipelines or recent deep-learning paradigms, struggle to simultaneously satisfy the stringent demands of industrial scenarios: high accuracy on sub-millimeter flaws, insensitivity to texture-rich backgrounds, and real-time throughput on resource-constrained hardware. Although contemporary detectors have narrowed the gap, they still exhibit pronounced sensitivity–robustness trade-offs, particularly in the presence of scale-varying defects and cluttered surfaces. To address these limitations, we introduce MBY (MBDNet-Attention-YOLO), a lightweight yet powerful framework that synergistically couples the MBDNet backbone with the YOLO detection head. Specifically, the backbone embeds three novel components: (1) HGStem, a hierarchical stem block that enriches low-level representations while suppressing redundant activations; (2) Dynamic Align Fusion (DAF), an adaptive cross-scale fusion mechanism that dynamically re-weights feature contributions according to defect saliency; and (3) C2f-DWR, a depth-wise residual variant that progressively expands receptive fields without incurring prohibitive computational costs. Building upon this enriched feature hierarchy, the neck employs our proposed MultiSEAM module—a cascaded squeeze-and-excitation attention mechanism operating at multiple granularities—to harmonize fine-grained and semantic cues, thereby amplifying weak defect signals against complex textures. Finally, we integrate the Inner-SIoU loss, which refines the geometric alignment between predicted and ground-truth boxes by jointly optimizing center distance, aspect ratio consistency, and IoU overlap, leading to faster convergence and tighter localization. Extensive experiments on two publicly available steel-defect benchmarks—NEU-DET and PVEL-AD—demonstrate the superiority of MBY. Without bells and whistles, our model achieves 85.8% mAP@0.5 on NEU-DET and 75.9% mAP@0.5 on PVEL-AD, surpassing the best-reported results by significant margins while maintaining real-time inference on an NVIDIA Jetson Xavier. Ablation studies corroborate the complementary roles of each component, underscoring MBY’s robustness across defect scales and surface conditions. These results suggest that MBY strikes an appealing balance between accuracy, efficiency, and deployability, offering a pragmatic solution for next-generation industrial quality-control systems.

## 1. Introduction

Driven by progressive industrial automation, the steel sector now stands as a linchpin of worldwide infrastructure; its quality directly governs structural safety and operational longevity [1]. Central to this quality assurance pipeline is surface defect detection [2], since imperfections—pores, cracks, scratches, pits, and decarburization—frequently originate from raw-material contaminants, process deviations, or equipment malfunctions [3]. Beyond cosmetic degradation, these anomalies erode mechanical integrity and corrosion resistance: micro-cracks and pores act as fracture nuclei during subsequent forming or service, whereas scratches and pits undermine coating adhesion and surface-treatment efficacy. From a metallurgical standpoint, many surface defects originate from microstructural mechanisms such as grain boundary decohesion, inclusion-induced stress concentration, or localized plastic deformation. These processes lead to macroscopic manifestations like cracks, pores, and patches—patterns that are directly captured in visual inspection and form the basis of defect recognition in computer vision models.

Surface defect detection is critical in both hot-rolled and cold-rolled steel production, as each involves distinct defect characteristics and detection challenges. Hot-rolled steel typically suffers from scale, cracks, and inclusions due to exposure to high temperatures and intense deformation forces, while cold-rolled steel is more susceptible to scratches, dents, and irregularities caused by roll wear or lubrication inconsistencies. Effective detection systems must be tailored to these domain-specific conditions. In this study, we focus specifically on hot-rolled steel to address its characteristic defect patterns and industrial requirements.

Steel surface anomaly detection has crystallized into two paradigms: traditional image-processing pipelines and deep-learning-based intelligent systems [4]. Classical techniques hinge on operator expertise and hand-crafted descriptors, thereby failing to reconcile the modern production mandate for simultaneous throughput and sub-millimeter precision [5]. With the advent of Machine Learning and Cognitive Computing—most notably deep-learning—data-driven detectors have become the prevailing direction. Current detectors fall into two camps: two-stage models like R-CNN [6] give high accuracy but run slowly because they first generate region proposals and then refine them, while single-stage models like YOLO [7,8,9] and SSD [10] skip the proposal step for speed but lose a bit of accuracy. Since the original YOLOv1 was introduced in 2016, the YOLO series has undergone continual improvement. YOLOv3 enhanced small-object detection via multi-scale predictions. YOLOv4 introduced CSP modules and Mish activation to boost accuracy and speed. YOLOv5 streamlined training pipelines and deployment. YOLOv7 integrated E-ELAN and re-parameterization for better real-time accuracy. Most recently, YOLOv8 refined architecture design and loss functions for improved robustness and performance. These developments have solidified YOLO’s dominance in efficient, high-precision object detection, especially in industrial scenarios. However, static Feature Pyramid Networks (FPNs), as widely used in prior detection frameworks, rely on fixed top-down fusion pathways and uniform weighting schemes, which limits their ability to adapt to complex defect distributions. In industrial scenarios where defect sizes vary drastically and backgrounds exhibit strong interference, such rigid structures often fail to capture informative scale-specific features. This limitation motivates the use of Dynamic Align Fusion (DAF), which adaptively aligns and aggregates multi-scale features based on spatial and semantic cues. Moreover, steel defects in real production are irregular and often sub-millimeter, making precise localization difficult. Reliable detectors must therefore (1) extract hierarchical features that capture subtle cues and (2) remain stable across varying backgrounds, textures, and lighting; both demands place significant constraints on robustness.

Consequently, CNN-based object detection has emerged as the dominant frontier in steel surface anomaly identification. To evaluate detection performance, we adopt standard mean Average Precision (mAP) metrics at IoU thresholds of 0.5 (mAP@0.5) and the averaged range from 0.5 to 0.95 with 0.05 intervals (mAP@0.5–0.95), which are widely used in defect detection to assess both detection sensitivity and localization accuracy. Zhao et al. [11] propose RDD-YOLO, an augmented YOLOv5 variant that enlarges the effective receptive field via Res2Net blocks, reinforces feature reuse through a Dual Feature Pyramid Network (DFPN), and disentangles classification and regression by adopting a decoupled detection head. On NEU-DET and GC10-DET, RDD-YOLO improves mAP by 4.3% and 5.8%, respectively, without compromising inference speed. Complementarily, Sunkara et al. [12] introduce YOGA, a lightweight architecture that marries efficient feature learning with multi-scale attention. A CSPGhostNet backbone and AFF-PANet fusion layer jointly condense parameters and computation, while a two-stage refinement pipeline and grouped convolutions further curb FLOPs. Crucially, a localized attention mechanism in the neck selectively amplifies small-defect cues. On the COCO validation set, YOGA elevates AP by 15% relative to YOLOv5 while trimming computational cost by 29% and parameter count by 23%, underscoring its suitability for resource-constrained edge deployment.

In addition to CNN-based approaches, transformer-based architectures and learning-based methods have recently gained increasing attention in the field of surface defect detection. Transformer-based models are particularly effective in capturing long-range dependencies and complex contextual features. For instance, Zhou et al. [13] proposed the GDALR model, which integrates a dual-branch transformer structure to simultaneously capture both local and global defect features. The model leverages a pure transformer branch for short-range dependency modeling through token detail aggregation, while utilizing pixel shuffle operations to enhance salient global representations across feature scales.

Similarly, Sun et al. [14] introduced SDD-DETR, which is the first work to apply the Detection Transformer (DETR) framework to aero-engine blade surface defect detection. The model incorporates two lightweight modules—a progressive feature input multi-scale deformable attention module (PFI-MSDA) and a lightweight feedforward network (LW-FFN)—to reduce computational costs while maintaining detection accuracy. Specifically, PFI-MSDA hierarchically reduces token inputs to the self-attention layers, and LW-FFN streamlines the network’s multilayer perceptron, making the model more efficient without sacrificing detection performance.

Huang et al. [15] developed ACViT, an adaptive cross transformer integrated with contrastive learning, to improve the detection of small-scale defects. ACViT adopts a meta-learning framework to enhance generalization across varying defect detection tasks. The model also incorporates self-supervised contrastive learning to improve feature distinctiveness, thereby increasing robustness against diverse defect types [16].

Beyond transformer-based approaches, self-supervised learning and few-shot learning methods have also been explored to address challenges arising from limited labeled data. These methods enable models to learn generalized feature representations that can be adapted to novel categories or small sample scenarios. For example, Su et al. proposed a surface defect detection method based on few-shot learning [17]. Their model enhances the traditional Faster R-CNN framework by incorporating deformable convolutions into the ResNet101 and FPN backbone, followed by object pyramid construction to enrich feature scales of small samples. Furthermore, the model applies contrastive learning on Region of Interest (RoI) features to improve compactness and reduce misclassification, thereby improving detection performance on rare defect categories.

While these advanced methods exhibit strong detection performance, their high computational costs and complex architectures often limit their applicability in real-time industrial scenarios. Consequently, YOLO-based detectors remain preferable in many practical applications due to their fast inference and relatively low computational complexity. Nevertheless, most existing YOLO-based methods still face challenges in detecting small defects and maintaining robustness under complex backgrounds, which motivates our work in this paper.

YOLO algorithms have become the predominant choice for industrial defect detection due to their ability to deliver real-time detection combined with strong classification accuracy. For instance, recent research applying YOLO to product defect detection has demonstrated that YOLO-based methods can achieve efficient inference while maintaining high detection accuracy, greatly enhancing quality inspection throughput and reliability [18]. Meanwhile, although transformer-based detectors typically achieve superior precision, their higher computational cost and longer inference latency often limit their feasibility in industrial real-time applications. Therefore, striking an effective balance between detection accuracy and computational efficiency remains a critical challenge. Our proposed MBY method builds upon YOLO’s strengths by integrating an efficient backbone and attention mechanisms to improve sensitivity and robustness without sacrificing inference speed.

Shi et al. [19] refine Faster R-CNN by grafting a ConvNeXt backbone and plugging a CBAM block to foreground defect cues while suppressing background clutter; anchor priors are further optimized via k-means clustering. The resulting model attains 80.78% mAP on NEU-DET, surpassing YOLOv5 by 1.5% and vanilla Faster R-CNN by 8.4%, while sustaining 26 FPS inference. Meng et al. [20] surgically replace the SPPF module in YOLOv8n, yielding consistent accuracy and latency gains on NEU-DET, thereby corroborating its efficacy for defect detection. Liu et al. [21] propose EZS-YOLOv10, a zero-shot extension of YOLOv10 tailored for micro-defects. Adaptive multi-scale fusion and a refined anchor generator are coupled with a Region Feature Synthesis network for unseen-class generalization. On COCO, EZS-YOLOv10 registers +1.5% mAP overall and +2.7% on small objects; analogous gains (+2.7% mAP) are observed on VisDrone. Guo et al. [22] embed the Transformer-inspired TRANS module into YOLOv5, yielding MSFT-YOLO. Multi-scale features are harmonized via BiFPN, empowering the detector to penetrate background noise and resolve minute flaws. The model elevates NEU-DET mAP by 7%. Tang et al. [23] present a YOLOv5n variant that interlaces an attention mechanism within the backbone and refines training dynamics via a redesigned loss. Consequently, recall rises by 8.3% and AP by 3.6% on steel-strip data, while training time is slashed by 44%. Jiang et al. [24] craft YOLOv8n-SDEC, substituting SPPF with SPPCSPC for richer multi-scale context, integrating DCNv2 and CARAFE for deformable receptive fields and content-aware upsampling, and adopting EIoU loss for tighter regression. The model records a 3.3% mAP boost on NEU-DET. Zhong et al. [25] augment YOLOv5 with an OSA-C3 module for deeper feature transformation, a BiC-PAN for cross-scale fusion, and Coordinate Attention for precise localization. The enhanced detector reaches 79.1% mAP on NEU-DET (+2.6% vs. baseline) without sacrificing speed. Zhao et al. [26] introduce AMFF, an attention-guided multi-scale fusion plug-in composed of SEAM (self-enhanced dilated-attention) and CEAM (cross-layer attention). When inserted into FPN/PAFPN, AMFF consistently elevates both accuracy and real-time performance across defect detection benchmarks.

To address the aforementioned challenges, we present MBY (MBDNet-Attention-YOLO), a purpose-built steel surface defect detector that synergizes a lightweight dual-backbone architecture with targeted attention mechanisms. Concretely, we first devise MBDNet, an efficient feature-extraction backbone that interleaves HGStem, Dynamic Align Fusion, and C2f-DWR blocks to harvest and refine multi-scale representations while curbing computational overhead. Second, the neck is augmented with the MultiSEAM module, which jointly re-calibrates spatial and channel dependencies across scales, thereby sharpening sensitivity to microscopic flaws embedded in textured backgrounds. Third, we adopt an improved Inner-SIoU loss that tightens the geometric alignment between predicted and ground-truth boxes, accelerating convergence and elevating localization precision. Extensive evaluations on the public NEU-DET and PVEL-AD benchmarks confirm that MBY surpasses contemporary baselines in both accuracy and recall without sacrificing real-time capability.

The principal contributions are summarized below:

1. We introduce MBY, a novel steel-defect detector that fuses the MBDNet backbone with the YOLO framework, attaining state-of-the-art accuracy while preserving inference efficiency.
2. We create the MBDNet architecture, a compact yet powerful backbone that leverages HGStem, Dynamic Align Fusion, and C2f-DWR to enrich multi-scale feature extraction.
3. We integrate the MultiSEAM module within the detection head to fuse cross-scale spatial-channel information, bolstering robustness against cluttered backgrounds and minute defects.
4. We devise Inner-SIoU loss to enhance bounding box regression accuracy and training stability, further improving overall model robustness.

## 2. Proposed Method

### 2.1. Backbone Network: MBDNet

**Figure 1:**
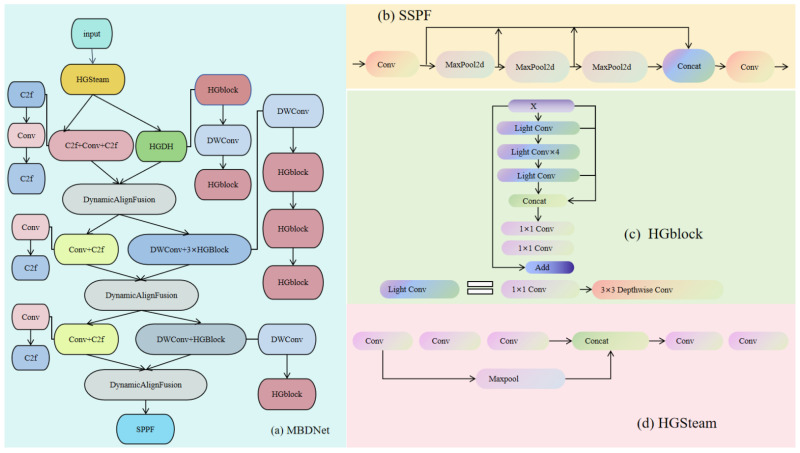
(**a**) MBDNet: dual-backbone network architecture diagram presents the overall architecture of the dual-backbone network, including the inputs, processing stages, and fusion module of the two backbone networks. (**b**) SPPF: SPPF module structure diagram provides a detailed description of the internal structure of the SPPF module, including multi-scale pooling and feature concatenation. (**c**) HGblock: HGblock module structure diagram displays the structure of the HGBlock module, including convolutional layers, lightweight convolution, and residual connections. (**d**) HGSteam: HGSteam module structure diagram describes the multi-stream feature processing and fusion mechanism of the HGStream module.

In this study, we propose a novel backbone architecture named MBDNet, characterized by a dual-backbone framework designed to improve both feature extraction and integration. The comprehensive structure of the proposed network is depicted in Figure 1. Conventional convolutional neural networks (CNNs) frequently encounter difficulties in efficiently capturing multi-scale features, especially when addressing complex defect recognition challenges, which consequently hinders the comprehensive efficacy of the model [27].

To address this, we design two specialized modules: HGStem for low-level feature extraction and C2f for high-level feature fusion, both significantly improving the network’s representational capacity.

To further boost computational efficiency, we integrate the Depthwise Separable Convolution (DWConv) module [28]. DWConv decomposes standard convolutions into depthwise and pointwise operations, drastically reducing computational cost while preserving the ability to extract rich multi-scale feature information [29]. Additionally, to strengthen feature fusion capabilities, we incorporate the Dynamic Align Fusion module, which aligns and merges feature maps across different scales, thereby enhancing the model’s multi-scale perception [30]. Ultimately, the Spatial Pyramid Pooling Fast (SPPF) module is integrated to enhance the perceptual domain, ergo boosting the network’s ability to detect objects of diverse sizes. To avoid conflicting gradient updates, the dual backbones share a unified loss through the fused features in the Dynamic Align Fusion module. This joint supervision ensures consistent gradient flow to both branches, harmonizing their learning despite focusing on different feature levels [31]. Consequently, the model achieves stable and coordinated optimization across the dual-backbone structure.

In summary, the core components of MBDNet include HGBlock, DWConv, Dynamic Align Fusion, and SPPF, collectively enabling efficient and robust multi-scale feature extraction and fusion.

**Figure 2:**
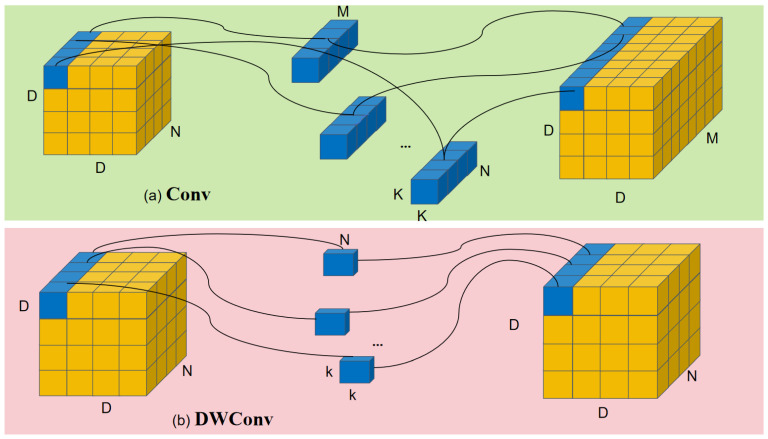
Schematic diagrams of different convolution operations: (**a**) Standard convolution (Conv); (**b**) Depth-wise convolution (DWConv).

In tasks involving small-object detection, standard convolutions, shown in Figure 2a, collect much redundant detail when targeting small objects, raising computation. We therefore replace them with Depthwise Separable Convolution (DWConv). The DWConv process, illustrated in Figure 2b, decomposes convolution into two distinct stages. Initially, depthwise convolution is applied independently on each input channel, producing a reduced feature map, denoted as $X_{a}$.

Subsequently, a simpler linear operation is conducted: group convolution is performed channel-wise, instead of the cross-channel convolution used in standard methods, resulting in a redundant feature map, denoted as $X_{b}$.

In the end, these two feature maps, denoted as $X_{a}$ and $X_{b}$, are merged to produce the final output feature map with depth, which can be expressed as

(1) $$X_{a}=X⊗W_{1}$$

(2) $$X_{b}=X⊗W_{2}$$

(3) $$Y=X_{a}⊕X_{b}$$

where, X refers to the feature map at the input layer, while $W_{1}$ denotes the standard convolution kernel and $W_{2}$ denotes the group convolution kernel. ⊗ stands for the standard convolution operation, and ⊕ indicates concatenation along the channel dimension [32].

### 2.2. Dynamic Align Fusion (DAF)

**Figure 3:**
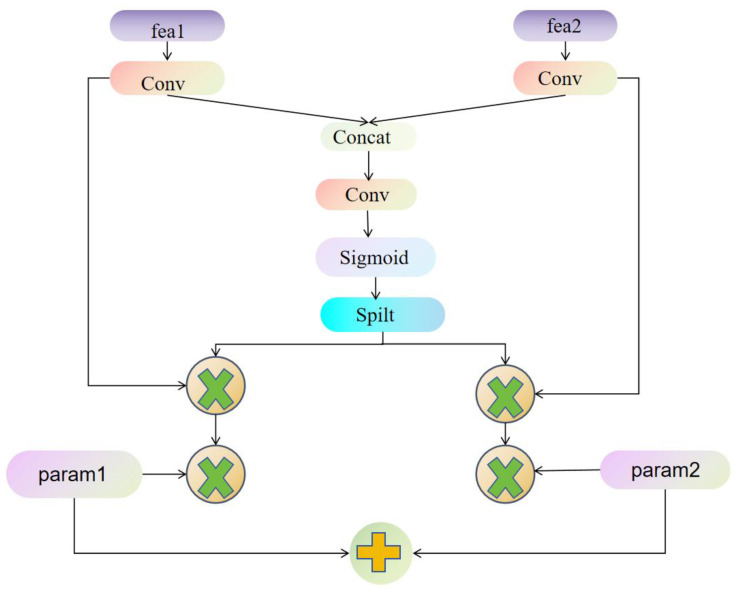
Network architecture of the Dynamic Align Fusion (DAF) mechanism.

To reduce feature misalignment between backbones, we propose Dynamic Align Fusion (DAF) [33]. Since the feature maps differ in channels, stride, and receptive field, simple concatenation or weighted averaging lowers quality [34]. DAF addresses this by implementing feature alignment, adaptive weighting, and channel-wise modulation, enabling more precise fusion and thereby boosting object detection network performance [35]. The working principle of DAF is depicted in Figure 3. This approach is compatible with various architectures such as YOLOv8, EfficientDet [36], and PP-YOLO [37], enhancing detection accuracy while keeping additional computational costs low.

In the DAF structure [33], 1×1 convolutions are first applied to the input features $X=\{x_{1},x_{2}\}$ for channel alignment, ensuring that they can be fused within a common feature space. Since *x*_1_ and *x*_2_ may originate from different backbones and thus have mismatched channel dimensions, the following adjustments are required:

(4) $$\left\{\begin{array}{c} {\hat{x}}_{1}=Conv_{1\times 1}\left(x_{1}\right)\\ {\hat{x}}_{2}=Conv_{1\times 1}\left(x_{2}\right) \end{array}\right.$$

Here, ${W_{1}}^{\left(1\times 1\right)}$ and ${W_{2}}^{\left(1\times 1\right)}$ are 1×1 convolution weights used for channel transformation to align *x*_1_ and *x*_2_ along the channel dimension. This operation balances the information distribution across channels, providing a foundation for subsequent fusion.

After channel alignment, the aligned features ${\hat{x}}_{1}$ and ${\hat{x}}_{2}$ are concatenated. To make the fusion process more adaptive, the Dynamic Align Weight (DAW) mechanism is introduced. Specifically, a 3×3 convolution is performed on the concatenated features to capture high-level integrated information:

(5) $$x_{concat}=\left[{\hat{x}}_{1},{\hat{x}}_{2}\right],\;W_{align}=\sigma \left({Conv}_{3\times 3}(x_{conact})\right)$$

where, ${Conv}_{3\times 3}$ denotes a 3×3 convolutional layer utilized to derive the fused features from the concatenated input.

$\sigma \left(\cdot \right)$ represents the logistic function, which scales the weights to lie within the range (0, 1), enabling adaptive normalization of features from different sources. Subsequently, the output $W_{align}$ is partitioned along the channel axis into two separate dynamic weight tensors, $w_{1}$ and $w_{2}$:

(6) $$w_{1},w_{2}=split(W_{align},2)$$

Ultimately, the mechanism of DAF dynamically adjusts the role and degree of contribution of each input feature in the overall process through reasonable weight allocation; as indicated in Equation (7), its core purpose is to enable smoother and more coordinated mutual fusion among features generated by different backbone networks, thereby achieving a more optimal synergistic effect.

(7) $$\left\{\begin{array}{c} \tilde{x_{1}}=w_{1^{⊙}}{\hat{x}}_{1}\\ \tilde{x_{2}}=w_{2^{⊙}}{\hat{x}}_{2} \end{array}\right.$$

where, ⊙ represents element-wise multiplication, where dynamic weights are used to assign a weighted contribution to the input features, ensuring that the effective information from different sources is more aligned with the fusion requirements. 

However, relying solely on dynamic weights may lead to the excessive suppression of certain feature paths. Therefore, we further introduce Learnable Channel Weights to address this issue. $\lambda_{1}$ and $\lambda_{2}$ are used to optimize the ratio of the final fused features, allowing the network to be designed to adaptively modify the impact of various routes according to the input representations:

(8) $$y={Conv}_{1\times 1}(\lambda_{1}\cdot \tilde{x_{1}}\;+\lambda_{2}\cdot \tilde{x_{2}})$$

where, $\lambda_{1}$ and $\lambda_{2}$ are trainable parameters, initialized to 0.5, and automatically optimized during training to enable the model to learn the optimal feature fusion ratio. To prevent $\lambda_{1}$ and $\lambda_{2}$ from experiencing gradient explosion or numerical instability during training, DAF imposes the following constraints:

(9) $$\lambda_{i}=clip\left(\left|\lambda_{i}\right|,1.0\right)\cdot sign\left(\lambda_{i}\right),i\in \{1,2\}$$

These constraints keeps channel weights in a stable range, safeguarding model consistency and generalization. Finally, we use a 1×1 convolution to adapt the features for downstream detection tasks.

### 2.3. C2f-DWR Module

Although YOLOv8’s C2f (Cross Stage Partial Feature Fusion) module effectively propagates information across layers and balances accuracy with efficiency [38], the standard instantiation proves inadequate for steel surface anomalies. Edge cracks, pits, scratches, and decarburized regions exhibit extreme scale variance, irregular topology, and blurred boundaries; under such conditions, C2f’s fixed receptive fields struggle to harvest sufficient context, leading to missed detections or spurious alarms. To remedy this limitation, we introduce C2f-DWR, a plug-in replacement that embeds Dilated Residual (DWR) units [39] into the original C2f framework. By systematically enlarging receptive fields with dilated convolutions while preserving residual connectivity, C2f-DWR enriches multi-scale representations and sharpens responses to defects of disparate shapes and sizes without increasing computational overhead.

**Figure 4:**
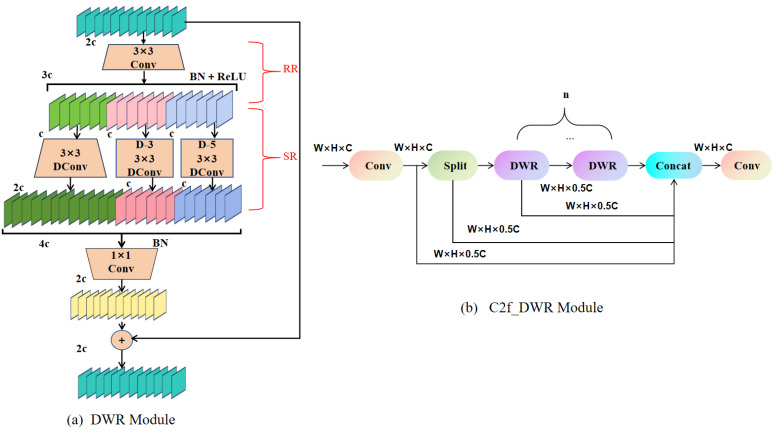
DWR module and C2f-DWR architecture diagram.

The structural design of the DWR module is illustrated in Figure 4a. This module adopts a dual-branch structure consisting of Region Residual (RR) and Semantic Residual (SR) components, aimed at enhancing feature extraction and enabling the model to achieve enhanced proficiency in managing defects that exhibit a wide range of dimensions.

Initially, the input features are processed with a 3×3 standard convolution. Afterward, the framework incorporates a normalization technique based on batch statistics, which is succeeded by an element-wise thresholding operation to introduce nonlinearity into the data flow, thus enabling the model to perform both normalization and nonlinear transformation effectively. This part belongs to the RR branch, which is primarily responsible for extracting local detail information, enabling the model to better recognize small defects.

Next, the features are passed to the SR branch for further feature extraction. Subsequent to the application of 3×3 depthwise convolution (DConv), the feature maps are subjected to a pair of 3×3 depthwise convolutions, each characterized by distinct dilation rates, thereby facilitating multi-scale feature extraction. Specifically, one branch employs a dilation rate of 3 (denoted as D-3), while the other utilizes a dilation rate of 5 (denoted as D-5). This dual-branch approach effectively expands the receptive field, thereby facilitating the model’s capacity to assimilate a more expansive ambit of contextual intelligence.

Finally, the features from both branches are concatenated, followed by batch normalization (BN) for data adjustment and a dimensionality-reducing 1×1 convolutional layer is utilized to alleviate computational burden. Thereafter, the resultant feature map is amalgamated with the initial input features by means of a residual linkage. This process enables the network to conserve the pristine information, refine feature depiction, and augment detection precision.

As illustrated in Figure 4b, the C2f-DWR unit interleaves dilated convolutions with progressive residual connections, systematically expanding the receptive field without enlarging kernels or deepening the stack. This dilation-centric design trades marginal memory for marked contextual gain, yielding richer multi-scale features at constant FLOPs. Consequently, the detector remains equally sensitive to microscopic scratches and expansive surface damage, enhancing both stability and cross-domain generalization.

### 2.4. Detect_MultiSEAM

Steel surface defects—scratches, pits, cracks, oxidation flecks, and peeling patches—span millimeter-scale to sub-pixel sizes and exhibit highly irregular geometries. Their visual signatures are further confounded by rolling textures, specular reflections, and acquisition noise, rendering robust detection non-trivial. 

**Figure 5:**
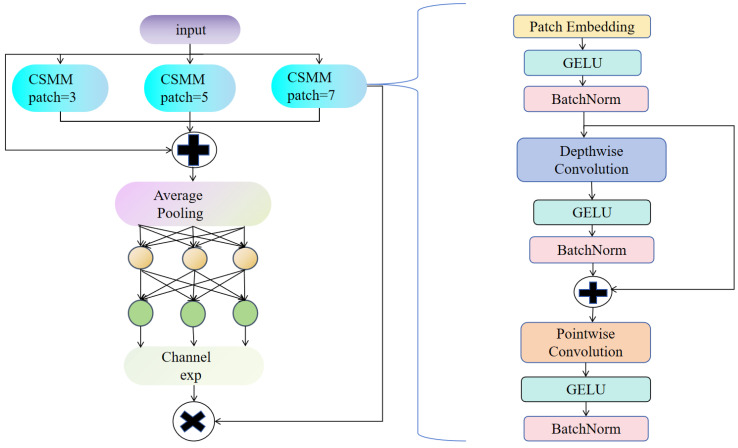
Architecture diagram of the MultiSEAM module.

To amplify discriminative cues, as shown in Figure 5, MultiSEAM module is embedded into the YOLOv8n detection head. MultiSEAM re-calibrates and fuses multi-scale backbone features, capturing both fine-grained textures and global defect morphology within a single unified representation. This enriched embedding markedly suppresses false positives and missed detections across defect taxonomies. Moreover, by consolidating contextual evidence under occlusion and uneven illumination, MultiSEAM endows the detector with heightened resilience and cross-scene generalisability.

To improve classification accuracy and generalization, label smoothing is applied in the classification head. This regularization technique adjusts hard target labels (0 and 1) to softer values (e.g., 0.1 and 0.9), reducing model overconfidence and enhancing robustness against noisy data. In our implementation, a label-smoothing factor of 0.1 is used, which effectively mitigates overfitting and improves detection performance during training.

MultiSEAM improves detection accuracy by strengthening feature extraction in unoccluded regions and alleviating the performance degradation caused by object occlusion. Its core component is the Channel and Spatial Mixed Module (CSMM), which processes input features using parallel convolution kernels of varying scales to more fully capture multi-scale information, allowing for more precise defect localization. Inside CSMM, depthwise-separable convolutions are coupled with residual links to cut computation yet retain feature fidelity. Since handling channel information individually might overlook inter-channel correlations, a 1 × 1 convolution is additionally employed to integrate channel features and enhance feature interactions. The processed features then undergo global average pooling to reduce spatial dimensions, minimizing redundant information and boosting feature stability. Subsequently, a dual-layer fully connected network further integrates channel information, thereby reinforcing the interconnections among distinct features. Finally, channel expansion operations integrate multi-scale information effectively, significantly reducing detection errors caused by occlusion or complex background interference.

### 2.5. Inner-SIoU

Steel surface defects are minute and morphologically diverse, often yielding indistinct boundaries that undermine detection accuracy. The canonical Complete IoU (CIoU) loss frequently introduces false positives and negatives during box regression, degrading precision and hindering convergence. To remedy this, we adopt Inner-SIoU (Inner Shape Intersection over Union), a ratio-scaled extension of SIoU that adjusts an auxiliary bounding box to stabilize gradient flow and sharpen directional supervision. 

CIoU’s supervision is limited to overlap, center distance, and aspect ratio. By contrast, Inner-SIoU enhances directional awareness, leading to tighter defect boundaries on complex steel surfaces and significantly faster convergence.

Inner-SIoU dynamically adjusts predicted bounding boxes based on the shape characteristics of the internal overlap with ground-truth boxes to improve alignment precision. When the predicted box is larger than the ground-truth box, Inner-SIoU identifies and reduces the excess regions by selectively adjusting width or height to better fit the ground-truth boundary, avoiding distortions caused by uniform scaling. 

Conversely, if the predicted bounding box is undersized, it expands the box’s dimensions and adjusts its angle toward the uncovered ground-truth regions, ensuring accurate filling without over-extension or under-extension. This shape-aware adjustment enables more precise localization compared to traditional IoU metrics.

**Figure 6:**
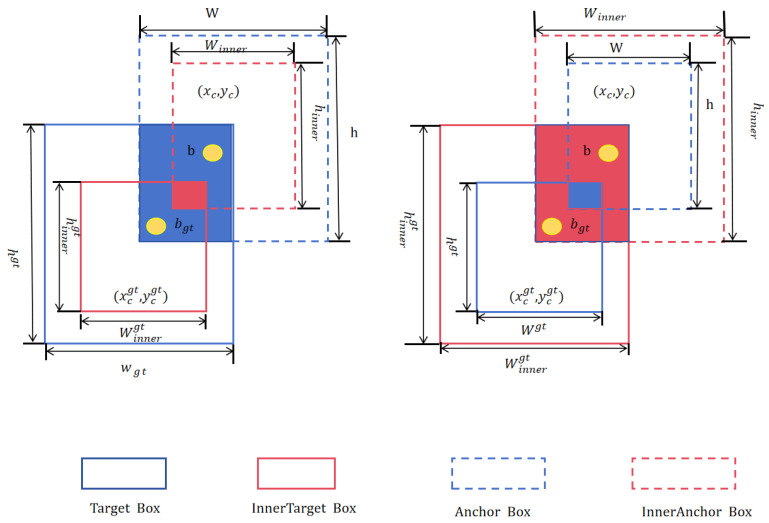
Network architecture diagram of SIoU.

The Inner-SIoU loss modulates the auxiliary bounding box size via the scale factor, making it adaptable to various datasets and detectors, thereby addressing limitations in detection accuracy of existing methods. Specifically, as illustrated in Figure 6, the target box (TB) and anchor box (Anchor Box) are denoted as $b_{gt}$ and b, respectively, making it adaptable to various datasets and detectors, thereby addressing limitations in detection accuracy of existing methods. Specifically, as illustrated in Figure 6, the target box (TB) is represented as Box 1, while the anchor box is represented as Box 2. The central coordinates of Box 1 are $\left(x_{c}^{gt},y_{c}^{gt}\right)$, and those of Box 2 are $\left(x_{c},y_{c}\right)$. The respective widths and heights of Box 1 and Box 2 are $w_{gt}$, $h_{gt}$ and w, h. The scale factor $r_{ratio}$ generally varies between 0.5 and 1.5. When $r_{ratio}$ is less than 1, the auxiliary box is smaller than the ground-truth box, resulting in a larger IoU gradient magnitude for the auxiliary box compared to the original target box. This accelerates the convergence for samples with high IoU values. In contrast, when $r_{ratio}$ is greater than 1, the auxiliary box is larger than the ground-truth box, which broadens the regression range and facilitates learning for samples with low IoU values.

The ratio for Inner IoU is defined as follows:

(10) $$\left\{\begin{array}{c} b_{l}^{gt}=x_{c}^{gt}-\frac{W^{gt}\times r_{ratio}}{2}\\ b_{r}^{gt}=x_{c}^{gt}-\frac{W^{gt}\times r_{ratio}}{2} \end{array}\right.$$

where, $b_{l}^{gt}$ signifies the abscissa of the auxiliary bounding box’s left edge, and $b_{r}^{gt}$ indicates the abscissa of its right edge.

(11) $$\left\{\begin{array}{c} b_{t}^{gt}=y_{c}^{gt}+\frac{h^{gt}\times r_{ratio}}{2}\\ b_{b}^{gt}=y_{c}^{gt}+\frac{h^{gt}\times r_{ratio}}{2} \end{array}\right.$$

where, $b_{t}^{gt}$ signifies the perpendicular coordinate of the lower edge of the ancillary spatial localization frame, while $b_{b}^{gt}$ indicates the perpendicular coordinate of the upper edge.

(12) $$\left\{\begin{array}{c} b_{l}=x_{c}\frac{W\times r_{ratio}}{2}\\ b_{r}=x_{c}^{gt}+\frac{W\times r_{ratio}}{2} \end{array}\right.$$

(13) $$\left\{\begin{array}{c} b_{t}=y_{c}-\frac{h\times r_{ratio}}{2}\\ b_{b}=y_{c}^{gt}+\frac{h\times r_{ratio}}{2} \end{array}\right.$$

where, $b_{l}$ specifies the lateral position of the left extremity of the auxiliary anchor box; $b_{r}$ delineates the lateral position of the right extremity; $b_{t}$ denotes the vertical position of the lower extremity; and $b_{b}$ characterizes the vertical position of the upper extremity of the auxiliary anchor box.

(14) $$inter=(min\left(b_{r}^{gt},b_{r}\right)-max\left(b_{l}^{gt},b_{l}\right))\times (min\left(b_{b}^{gt},b_{b}\right)-max\left(b_{t}^{gt},b_{t}\right))$$

(15) $$union=\left(w^{gt}\times h^{gt}\right)\times {\left(ratio\right)}^{2}+\left(w\times h\right)\times {\left(ratio\right)}^{2}-inter$$

(16) $${IoU}^{inner}=\frac{inter}{union}$$

where, ${IoU}^{inner}$ corresponds to the Inner-IoU metric; inter indicates the area of overlap between the auxiliary delimiting box and the auxiliary reference box; union denotes the aggregate area encompassing both boxes.

Compared with CIoU, SIoU adds shape, center-distance, and angle terms to the basic IoU, yielding tighter bounding box alignment. The loss function is formulated as:

(17) $$L_{SIoU}=1-IoU+{\left(∆+\Omega \right)}^{2}$$

Here, the angle loss ∆ is calculated as follows:

(18) $$∆=\frac{1}{2}\sum_{w,h}\left(1-e^{-\gamma \rho }\right),\gamma =2-\Lambda$$

(19) $$\left\{\begin{array}{c} \rho_{x}={\left(w_{c}b_{x}-b_{gt}^{x}\right)}^{2}\\ \rho_{y}={\left(h_{c}b_{y}-b_{gt}^{y}\right)}^{2} \end{array}\right.$$

The angle Λ is calculated by Equation (20):

(20) $$\Lambda =\sin\left(2{sin}^{-1}\left(\frac{\sqrt{{\left(x_{gt}^{c}-x^{c}\right)}^{2}+{\left(y_{gt}^{c}-y^{c}\right)}^{2}}}{min(\left|x_{gt}^{c}-x^{c}\right|,\left|y_{gt}^{c}-y^{c}\right|)}\right)\right)$$

The computation of the shape loss Ω is articulated subsequently:

(21) $$\left\{\begin{array}{c} \Omega =\frac{1}{2}\sum_{w,h}\left(1-e^{-w_{t}}\right)\\ w_{t}=\frac{\left|w-w_{gt}\right|}{w_{max}(w,w_{gt})} \end{array}\right.$$

In summary, the Inner-SIoU loss amalgamates the SIoU loss and the Inner-IoU loss to refine bounding box regression within object detection frameworks. The Inner-SIoU loss function is formulated thusly:

(22) $$L_{Inner-SIoU}=L_{SIoU}+IoU-{IoU}_{inner}$$

### 2.6. Object Detection Network MBDNet-Attention-YOLO (MBY)

**Figure 7:**
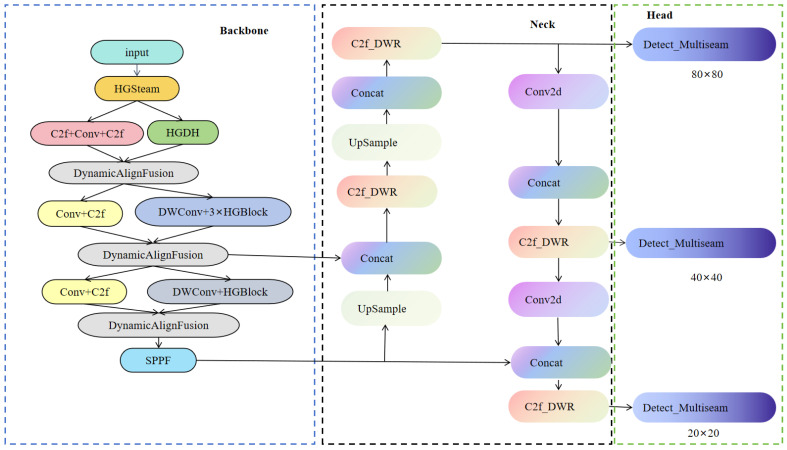
Architecture diagram of the MBY algorithm.

YOLOv8 begins by resizing every input image to a canonical resolution, after which a CNN backbone extracts multi-scale features. These features are fed into the detection head, which predicts bounding boxes, class scores, and objectness across three spatial resolutions; post-processing via NMS subsequently yields the final detection results. In this research, MBY detection algorithm is proposed (Figure 7), the proposed model is primarily composed of three essential parts: the backbone, the neck, and the head.

The backbone is entrusted with extracting critical image features and comprises several modules, including the HGStem module, C2f-DWR module, Dynamic Align Fusion (DAF) feature fusion module, DWConv convolution module, HGBlock module, and SPPF module. Collectively, these layers extract rich hierarchical features and strengthen the network’s representation.

The neck primarily integrates and refines feature information. It derives motivation from the Feature Pyramid Network (FPN) [40] and Path Aggregation Network (PANet) [41], enabling the integration of feature representations at multiple scales. This design markedly improves small-object recall and detection accuracy across scales.

The detection head is tasked with generating the ultimate detection results. Utilizing a decoupled detection head architecture, the model optimizes classification and regression tasks independently. Moreover, to boost small-object detection performance, an additional dedicated small-object detection layer is incorporated in the detection head, enabling MBY to reliably detect minute targets even in complex backgrounds.

## 3. Experiments

### 3.1. Data Description

**Figure 8:**
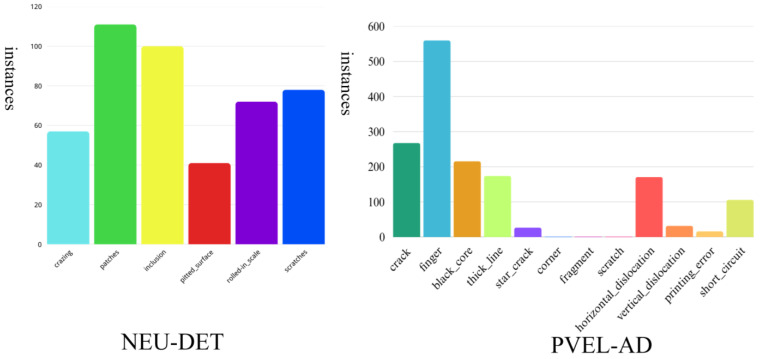
Category distribution in the two datasets.

The ongoing research utilizes the NEU-DET dataset provided by Northeastern University to evaluate the efficacy of the proposed network. The dataset encompasses six categories of steel surface defects: cracks (Cr), inclusions (In), patches (Pa), pitted corrosion (Ps), rust spots (Rs), and scratches (Sc). Each classification consists of 300 images, with each image exhibiting at least one defect. To further evaluate the generalization capability of the proposed framework, experiments are conducted on the PVEL-AD dataset. Table 1 summarizes the number of images in each category together with the training and validation splits. These datasets support the subsequent experimental investigation and result analysis. Figure 8 illustrates the allocation of defect types in both datasets.

### 3.2. Hyperparameter Settings

To verify the efficacy of the introduced MBY algorithm, all model architectures were realized using the PyTorch 1.8 framework on an NVIDIA GeForce RTX 3090 GPU. The hyperparameter configurations for the models are detailed in Table 2.

To evaluate the sustainability of our models, we measured the energy consumption per 1000 images processed using the NVIDIA System Management Interface (nvidia-smi) tool on the same hardware setup. The results indicated that the NEU-DET model consumed 9.72 Wh, while the PVEL-AD model consumed 9.74 Wh per 1000 images. These findings highlight that our models achieve high performance while maintaining excellent energy efficiency, which is crucial for sustainable computing.

### 3.3. Evaluation Metrics

In the realm of object detection, Precision (P) and Recall (R) are the standard metrics for evaluating object detection performance. They are defined as follows:

(23) $$Precision=\frac{TP}{TP+FP}$$

(24) $$Recall=\frac{TP}{TP+FN}$$

True positives (TPs) denote the quantity of accurately detected instances, false positives (FPs) denote the count of erroneously detected instances, and false negatives (FNs) denote the quantity of identified objectives that eluded detection by the model. To rigorously assess per-class performance, we adopt Average Precision (AP), computed as the area under the Precision–Recall (PR) curve, where Recall spans the horizontal axis and Precision the vertical. The area beneath this curve corresponds to the AP. The mean Average Precision (mAP) is ascertained by computing the mean of the AP values of all categorical divisions. The equation is articulated thusly:

(25) $$AP=\int_{0}^{1}P\left(R\right)dR$$

(26) $$mAP=\frac{1}{K}\sum_{i=1}^{K}AP_{i}$$

where, *K* signifies the total number of object classes within the dataset. The mAP serves as an indicator of the model’s aggregate detection accuracy spanning all genres. A superior mAP value is indicative of enhanced detection efficacy of the model across all categories.

### 3.4. An Analytical Comparison of Backbone Networks

To empirically verify the effectiveness of the proposed MBDNet backbone, we conduct comparative experiments on the NEU-DET and PVEL-AD datasets, evaluating it against several representative backbone architectures. Table 3 summarizes the performance of YOLOv8, HGNetV2 [42], MobileNetV4 [43], and EfficientFormerV2 [44], and the performance of our proposed MBDNet is assessed with respect to mAP@0.5, mAP@0.5–0.95, recall, and computational complexity (GFLOPs). The findings indicate that MBDNet attains an optimal trade-off between detection performance and computational cost on both datasets.

On the NEU-DET dataset, HGNetV2 integrates Graph Convolutional Networks to enlarge the receptive field and enhance global context aggregation. This refinement elevates recall to 75.1%, an absolute gain of 2.3 percentage points over the baseline, while the overall computational load remains modest at 6.9 GFLOPs. However, its feature representation remains limited, yielding an mAP@0.5–0.95 of only 52.0, indicating room for improvement in overall detection accuracy. MobileNetV4 leverages depthwise separable convolutions to optimize computational efficiency, with a GFLOPs of 22.5, but achieves an mAP@0.5–0.95 of merely 53.6. These results suggest that both HGNetV2 and MobileNetV4 exhibit constrained feature extraction abilities in complex environments, struggling to balance efficiency and accuracy. EfficientFormerV2 combines TransformerV2 and CNN architectures, effectively balancing efficiency and accuracy; however, its recall slightly drops by 0.3% compared to the baseline, revealing deficiencies in cross-scale feature extraction and local information capture. Replacing the original backbone with MBDNet yields more substantial gains: mAP@0.5–0.95 rises by 2.7% and recall reaches 77.3%, at the cost of 11.5 GFLOPs. Thus, MBDNet delivers higher precision while keeping the computational overhead moderate, offering a favorable balance between accuracy and efficiency.

On the PVEL-AD dataset, performance disparities among backbones become more pronounced. HGNetV2 attains a respectable recall of 70.8% but an mAP@0.5–0.95 of only 47.6%. MobileNetV4, despite its lightweight design, suffers a steep decline to 42.7% mAP@0.5–0.95 and reduced recall, indicating poor adaptability to the complex defect classes in this dataset. EfficientFormerV2 achieves a modest improvement with an mAP@0.5–0.95 of 51.0%, but overall performance remains limited. In direct comparison, MBDNet attains 75.9% mAP@0.5, a clear margin over YOLOv8’s 64.6%, while keeping the computational load at a comparable 8.1 GFLOPs and further improving both recall and overall detection accuracy.

In summary, the proposed MBDNet backbone consistently delivers excellent detection performance with lower computational costs across both datasets, demonstrating its strong potential for defect detection tasks.

### 3.5. An In-Depth Comparative Investigation of Loss Functions

To mitigate the imprecise regression and slow convergence caused by the standard CIoU loss, we adopt Inner-SIoU for bounding box localization. Results comparing different loss functions are provided in Table 4.

On the NEU-DET dataset, the model using the Inner-SIoU loss achieved an mAP@0.5 of 82.5%, outperforming CIoU [45], WIoU [46], Inner-IoU [47], and EIoU [48] by 2.3%, 2.5%, 1.3%, and 1.1%, respectively. For the mAP@0.5–0.95 metric, it improved by 1.0% compared to CIoU, reaching 51.5%. Recall also rose to 73.1%, showing that Inner-SIoU cuts missed detections. Tests on PVEL-AD confirm its robustness: mAP@0.5 reaches 71.6%, ahead of CIoU, WIoU, Inner-IoU, and EIoU, while mAP@0.5–0.95 is 45.6% and recall is 66.7%. Although computational costs among these loss functions are comparable, the consistent performance gains of Inner-SIoU make it more suitable for defect detection scenarios demanding high precision [49].

**Figure 9:**
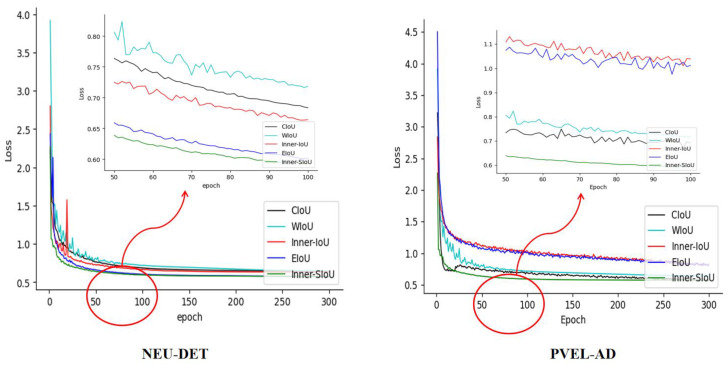
Loss function performance on NEU-DET and PVEL-AD.

Figure 9 illustrates the performance trends of different loss functions during training. Inner-SIoU demonstrates faster convergence on both datasets, with more stable loss reduction during training and validation phases. The trajectories of recall and mean Average Precision (mAP) metrics, including mAP@0.5 and mAP@0.5–0.95, manifest a progressive elevation, culminating in markedly enhanced performance levels. In summary, Inner-SIoU delivers consistent gains in both detection accuracy and recall across NEU-DET and PVEL-AD, confirming its practical value for steel surface defect detection [31].

### 3.6. Ablation Experiments

To systematically validate the contribution of every introduced module, we carried out extensive ablation studies on both the NEU-DET and PVEL-AD datasets, examining the incremental impact of each enhancement on overall detection performance. Table 5 and Table 6 summarize the results under various experimental settings, where MB-DAF, DWR, MultiSEAM, and Inner-SIoU represent the four key modules. A checkmark (“√”) signifies the incorporation of the module, whereas a cross (“×”) indicates its omission.

In the initial experiment, the baseline YOLOv8n model attained an mAP@0.5 of 80.2%, an mAP@0.5–0.95 of 50.5%, and a recall (R) of 72.8% on the NEU-DET dataset. In the subsequent experiment, the MB-DAF module was incorporated, thereby augmenting the model’s capacity to adapt to intricate steel surface defects. This change increased mAP@0.5 to 83%, confirming that the MB-DAF module strengthens detection across scales and suppresses background noise [49].

The third experiment integrated the DWR module, designed to improve the model’s multi-scale feature representation and small-object detection accuracy. This modification increased mAP@0.5 to 81.3%. The fourth experiment incorporated the MultiSEAM module, which strengthens multi-scale feature extraction and improves detection under complex backgrounds, elevating mAP@0.5 to 82.4%, mAP@0.5–0.95 to 51.3%, and recall as well [31].

In the fifth experiment, we replaced the default loss with Inner-SIoU, which leverages an internal bounding box to tighten the alignment between predicted and ground-truth boxes. This improved mAP@0.5 to 82.5% and mAP@0.5–0.95 to 51.5%. In experiment six, MultiSEAM and Inner-SIoU were combined to exploit their complementary strengths: MultiSEAM’s enhanced multi-scale feature extraction and Inner-SIoU’s refined bounding box regression. This synergy raised mAP@0.5 to 84.5%, mAP@0.5–0.95 to 54.0%, and recall to 79.3%, confirming the significant contribution of these modules in steel surface defect detection [50].

In the seventh experiment, MB-DAF, DWR, and MultiSEAM were jointly applied to further improve detection robustness in complex environments. The results indicated that the mAP@0.5 and mAP@0.5–0.95 values ascended to 85.8% and 56.2%, with the recall attaining a level of 79.7%. This validates that the integration of multiple optimization strategies can simultaneously enhance detection accuracy and model generalization [51].

To clarify the design philosophy behind the overall architecture, it is important to emphasize that these modules were systematically designed with complementary functionalities. MB-DAF improves shallow feature adaptation, DWR strengthens deeper-level feature fusion, MultiSEAM bridges scale gaps through spatial-channel synergy, and Inner-SIoU refines localization precision. Their cumulative gains are visible in the progressive performance increase across experiments.

Furthermore, the proposed method leverages a dual-backbone structure, which decouples low-level and high-level feature learning. One branch emphasizes fine-grained texture details critical for detecting small defects, while the other learns high-level semantic context useful for discriminating defect vs. background. Compared to single-backbone designs with enhanced multi-scale fusion, this separation allows more targeted optimization at each feature level, which contributes significantly to performance gains, especially in complex industrial scenarios.

This architecture design avoids unnecessary redundancy while preserving detection efficiency, as evidenced by competitive inference speeds and ablation improvements. The results demonstrate that each component meaningfully contributes to the final performance rather than introducing overfitting or excessive complexity.

Furthermore, experiments on the PVEL-AD dataset exhibited consistent significant improvements. Compared to the baseline YOLOv8n, the optimized MBY structure achieved an 11.3% increase in mAP@0.5, demonstrating the broad applicability and strong generalization potential of the proposed enhancements across diverse datasets.

Overall, the ablation study highlights the advantages of each improvement and further confirms their effectiveness.

It is worth noting that although our method achieves strong performance in terms of mAP@0.5, the mAP@0.5–0.95 metric remains relatively low on the PVEL-AD dataset. This phenomenon is commonly observed in small-object detection tasks, especially in industrial defect detection settings. The PVEL-AD dataset contains a large proportion of tiny, irregularly shaped defects that are extremely difficult to localize precisely under high IoU thresholds.

While mAP@0.5–0.95 provides a comprehensive evaluation of localization accuracy, its stringent requirements can overly penalize detection results on such challenging datasets. In practical industrial applications, detecting the presence of defects is often prioritized over precise pixel-level localization. Therefore, mAP@0.5 is widely regarded as a more suitable metric for evaluating detection performance in this context.

**Figure 10:**
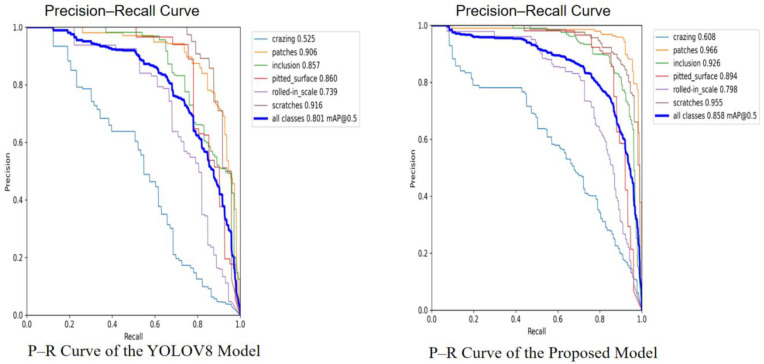
P–R curve of YOLOv8 and the proposed model on the NEU-DET dataset.

**Figure 11:**
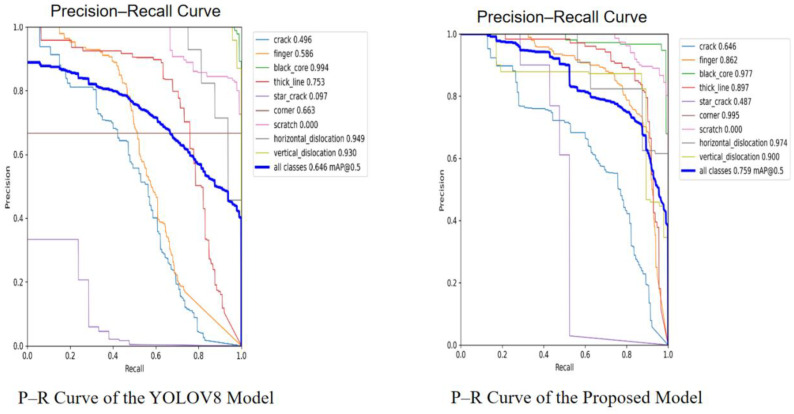
P–R curve of YOLOv8 and the proposed model on the PVEL-AD dataset.

Our method demonstrates consistent improvements in mAP@0.5 across datasets, which indicates its effectiveness in practical defect detection scenarios. This trade-off between detection sensitivity and localization precision reflects the inherent challenges of the task rather than a flaw in the model design. These patterns are clearly illustrated in the precision-recall curves: Figure 10 shows the comparison between YOLOv8 and our proposed model on the NEU-DET dataset, while Figure 11 presents the corresponding results on the PVEL-AD dataset. 

## 4. Comparison of Different Object Detection Models

To assess the efficacy of the proposed MBY model in steel surface defect detection tasks, empirical trials were executed utilizing two publicly available datasets: NEU-DET and PVEL-AD. The selected benchmark models include YOLOv5 [52], YOLOv6 [53], YOLOv8, YOLOv10, YOLOv11 [54], as well as other advanced detection models such as CAY [55], YOLOv7-BA [56], EFd-YOLOv4 [57], MS-YOLOv5s [58], PD-DETR [59], SSA-YOLO [60], and EDTNet [61]. All experiments were performed under consistent settings, with the specific detection metrics shown in Table 7 and Table 8.

In the initial experimental series, the YOLOv5 model attained mAP@0.5 of 84.5% and mAP@0.5–0.95 of 53.6% on the NEU-DET dataset. The second set of experiments introduced YOLOv6, which performed similarly to YOLOv5, with mAP@0.5 of 84.4% and a marginal enhancement in mAP@0.5–0.95. YOLOv8 and YOLOv10 demonstrated certain advantages in detection speed, but their overall accuracy did not meet expectations. YOLOv11, on the other hand, showed weaker performance, with mAP@0.5 of only 77.3%, indicating its subpar performance when dealing with complex textures.

In the sixth set of experiments, the proposed MBY model achieved superior performance compared to all other models with respect to detection correctness. On the NEU-DET dataset, the mAP@0.5 value ascended to 85.8%, while the mAP@0.5–0.95 metric climbed to 56.2% and recall reached 79.7%, with significant improvements across all metrics. This indicates that MBY excels in feature extraction, target localization, and small-defect recognition. On the PVEL-AD dataset, the MBY model also demonstrated excellent performance, with mAP@0.5 reaching 75.9%, outperforming models such as YOLOv8 and YOLOv10. Despite the improved detection accuracy, MBY’s computational cost remains at 8.1 G, comparable to YOLOv8, and significantly lower than YOLOv6’s 11.8 G, reflecting the model’s balanced performance between accuracy and efficiency.

**Figure 12:**
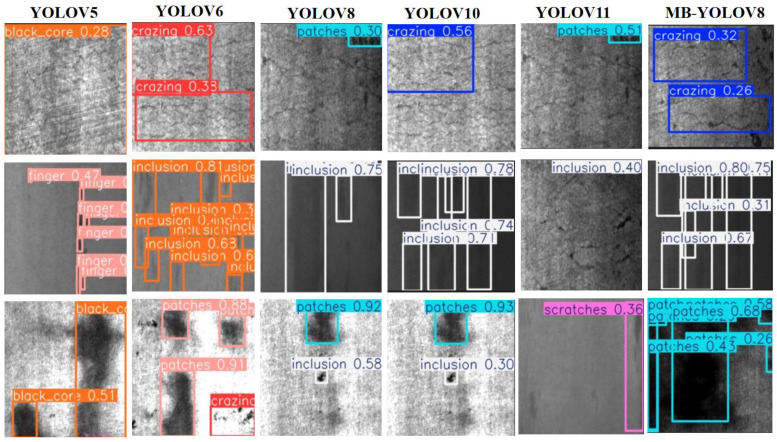
Comparative visualization of networks on NEU-DET dataset.

**Figure 13:**
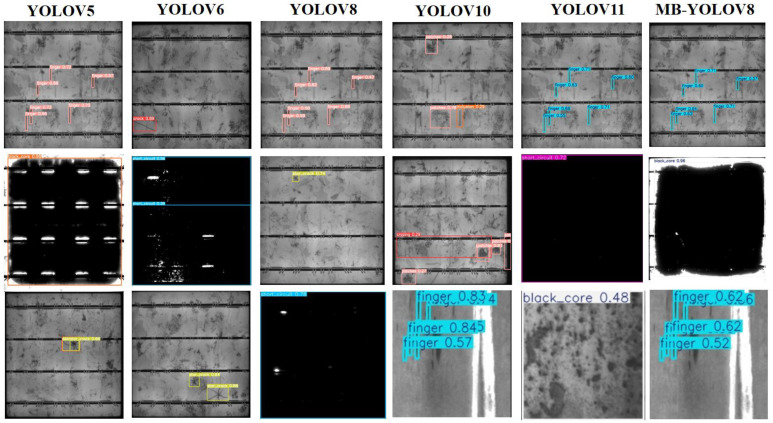
Comparison of visualization results of different networks on the PVEL-AD dataset.

Figure 12 and Figure 13 show the detection visualization results of MBY, YOLOv8, and YOLOv11 on two datasets. MBY demonstrates more stable performance in detecting small objects such as cracks, scratches, and pits, with more accurate bounding box localization and a significant reduction in false positives and false negatives.

It is worth emphasizing that the proposed model enhances detection performance and, concurrently, mitigates computational overhead to a notable extent. Overall, the empirical investigations within this segment validate the potency of the propounded enhancements and clearly highlight their advantages across different dimensions.

## 5. Conclusions

This study proposes MBY (MBDNet-attention-YOLO), a steel surface defect detector that first introduces the carefully engineered MBDNet backbone to markedly improve multi-resolution feature encoding and representation of defects. At the same time, the integration of the designed MultiSEAM module effectively improves feature adaptability across different scales and complex background conditions, particularly showing higher accuracy in handling small defects.

Next, to tackle the problem of inadequate bounding box regression precision, we introduce the Inner-SIoU loss function, optimizing the correspondence between predicted boxes and reference boxes, accelerating the convergence speed during training, and improving detection stability. Finally, we conducted comprehensive evaluations on the public NEU-DET and PVEL-AD datasets; experimental results show that the MBY algorithm achieved a detection accuracy of 85.8% on NEU-DET and 70.8% on PVEL-AD. Compared to mainstream single-stage detection algorithms such as YOLOv5, YOLOv6, and YOLOv8, MBY outperforms in multiple key metrics, demonstrating its strong adaptability and generalization capability in complex texture interference and small-object recognition.

To further propel this research, future endeavors will focus on the following three aspects: (1) refining the backbone network architecture to magnify the model’s capacity for representing multi-scale defect features; (2) combining different feature fusion strategies and attention mechanisms to further strengthen the model’s robustness in complex backgrounds; and (3) exploring the application of the MBY algorithm to other types of surface defect detection tasks.

## Figures and Tables

**Table 1:** Statistics of dataset categories and image counts.

Name	Number of Categories	Training Set	Validation Set	Test Set
NEU-DET PVEL-AD	6	1260	360	180
	12	3600	900	450

**Table 2:** Hyperparameter settings.

Name	NEU-DET	PVEL-AD
Optimizer	SGD	SGD
Learning rate	0.01	0.01
Weight decay factor	0.0005	0.0005
Batch size	32	32
Epoch	300	300
Image size	640 × 640	640 × 640
Energy consumption per 1000 images (Wh)	9.72	9.74

**Table 3:** Performance on NEU-DET and PVEL-AD with different backbones.

Dataset	Method	mAP@0.5	mAP@0.5–0.95	Recall	GFLOPs
NEU-DET	YOLOV8	80.2	50.5	72.8	8.1
	HGNetV2	81.3	52.0	75.1	6.9
	MobileNetV4	82.9	53.6	77.1	22.5
	EfficientFormerV2	81.6	51.0	72.5	9.4
	MBDNet	83.0	53.2	77.3	11.5
PVEL-AD	YOLOV8	64.6	44.1	60.2	8.1
	HGNetV2	69.1	47.6	70.8	6.9
	MobileNetV4	70.7	42.7	65.0	22.6
	EfficientFormerV2	67.6	51.0	72.5	9.4
	MBDNet	75.9	37.4	58.8	8.1

**Table 4:** Comparison of loss functions on NEU-DET and PVEL-AD datasets.

Dataset	Method	mAP@0.5	mAP@0.5–0.95	Recall	GFLOPs
NEU-DET	CIoU	80.2	50.5	72.8	8.1
	WIoU	80.0	50.1	72.5	8.2
	Inner-IoU	81.2	50.3	72.3	8.2
	EIoU	81.4	50.6	72.8	8.2
	Inner-SIoU	82.5	51.5	73.1	8.1
PVEL-AD	CIoU	64.6	44.1	60.2	8.1
	WIoU	70.8	50.3	69.0	8.1
	Inner-IoU	68.5	43.4	73.4	8.1
	EIoU	66.9	42.3	57.4	8.1
	Inner-SIoU	71.6	45.6	66.7	8.1

**Table 5:** Ablation experiments of MBY on the NEU-DET dataset.

Experiments	MB-DAF	DWR	MultiSEAM	Inner-SIoU	mAP@0.5	mAP@0.5–0.95	Recall	G
1	×	×	×	×	80.2	50.5	72.8	8.1
2	√	×	×	×	83.0	53.2	77.3	11.5
3	×	√	×	×	81.3	50.8	71.2	8.1
4	×	×	√	×	82.4	51.3	73.5	7.4
5	×	×	×	√	82.5	51.5	73.1	8.1
6	√	×	√	×	84.5	54.0	79.3	10.5
7	√	√	√	√	85.8	56.2	79.7	10.5

**Table 6:** Ablation experiments of MBY on the PVEL-AD dataset.

Experiments	MB-DAF	DWR	MultiSEAM	Inner-SIoU	mAP@0.5	mAP@0.5–0.95	Recall	G
1	×	×	×	×	64.6	44.1	60.2	8.1
2	√	×	×	×	68.4	42.5	77.3	9.9
3	×	√	×	×	68.8	44.8	60.2	8.0
4	×	×	√	×	71.4	48.8	69.6	7.3
5	×	×	×	√	71.6	45.6	66.7	8.1
6	√	×	√	×	69.3	36.6	71.2	9.7
7	√	√	√	√	75.9	37.4	58.8	8.1

**Table 7:** Comparison of detection performance for various single-stage algorithms on the NEU-DET dataset.

Models	mAP@0.5	mAP@0.5–0.95	GFLOPs
YOLOv5	84.5	53.6	7.1
YOLOv6	84.4	54.1	11.8
YOLOv8	80.2	50.5	8.1
YOLOv10	82.9	52.2	6.5
YOLOv11	77.3	50.9	6.3
CAY	79.9	45.6	\
YOLOV7-BA	74.8	38.8	\
EFd-YOLOv4	79.88	\	\
MS-YOLOv5s	80.5	\	\
SSA-YOLO	84.9	55.2	10.3
ETDNET	85.1	55.8	12.1
Ours	85.8	56.2	10.5

**Table 8:** Comparison of detection performance for various single-stage algorithms on the PVEL-AD dataset.

Models	mAP@0.5	mAP@0.5–0.95	GFLOPs
YOLOv5	69.5	44.8	7.1
YOLOv6	65.2	43.4	11.8
YOLOv8	59.2	37.7	8.1
YOLOv10	69.2	44.3	6.5
YOLOv11	69.3	45.9	6.3
PD-DETR	64.7	\	\
SSA-YOLO	70.3	40.1	10.3
ETDNET	71.2	39.8	12.1
Ours	75.9	37.4	8.1

## Data Availability

The original contributions presented in this study are included in the article. Further inquiries can be directed to the corresponding author.
